# *In Vitro* Characterization of a Biaryl Amide Anti-virulence Compound Targeting *Candida albicans* Filamentation and Biofilm Formation

**DOI:** 10.3389/fcimb.2018.00227

**Published:** 2018-07-10

**Authors:** Jesus A. Romo, Christopher G. Pierce, Marisol Esqueda, Chiung-Yu Hung, Stephen. P. Saville, Jose L. Lopez-Ribot

**Affiliations:** ^1^Department of Biology, South Texas Center for Emerging Infectious Diseases, The University of Texas at San Antonio, San Antonio, TX, United States; ^2^Department of Biology, University of the Incarnate Word, San Antonio, TX, United States

**Keywords:** *Candida albicans*, candidiasis, biofilms, filamentation, anti-virulence drugs

## Abstract

We have previously identified a small molecule compound, N-[3-(allyloxy)-phenyl]-4-methoxybenzamide (9029936), that exerts potent inhibitory activity against filamentation and biofilm formation by the *Candida albicans* SC5314 strain and represents a lead candidate for the development of anti-virulence approaches against *C. albicans* infections. Here we present data from a series of experiments to further characterize its *in vitro* activity and drug-like characteristics. We demonstrate the activity of this compound against a panel of *C. albicans* clinical isolates, including several displaying resistance to current antifungals; as well as against a set of *C. albicans* gain of function strains in key transcriptional regulators of antifungal drug resistance. The compound also inhibits filamentation and biofilm formation in the closely related species *C. dubliniensis*, but not *C. glabrata* or *C. tropicalis*. Combinatorial studies reveal the potential of compound 9029936 to be used together with currently available conventional antifungals. Results of serial passage experiments indicate that repeated exposure to this compound does not elicit resistance. Viability staining of *C. albicans* in the presence of high concentrations of compound 9029936 confirms that the compound is not toxic to fungal cells, and cytological staining using image flow cytometry analysis reveals that treatment with the lead compound affects hyphal length, with additional effects on cell wall and integrity of the membrane system. *In vitro* pharmacological profiling provides further evidence that the lead compound displays a safe profile, underscoring its excellent “drug-like” characteristics. Altogether these results confirm the potential of this compound to be further developed as a true anti-virulence agent for the treatment of *C. albicans* infections, including those refractory to treatment with conventional antifungal agents.

## Introduction

*Candida albicans* is an opportunistic pathogenic fungus commonly found in the majority of individuals as a commensal of the gastrointestinal and genitourinary tracts, as well as the skin, where it is controlled by host and microbial interactions (Romani et al., 2003, 2015; Kadosh and Lopez-Ribot, 2013; Salvatori et al., 2016). In healthy individuals, *C. albicans* is usually harmless, although it can develop into non-life threatening infections (i.e., vulvovaginal candidiasis) after heavy antibiotic treatment (Fidel, 1998). Moreover, an expanding number of immune-, as well as medically-compromised individuals such as those with catheters, stents, and prosthetics, are at risk of life threatening infections such as disseminated candidiasis (Brown et al., 2012). Although antifungal agents are available and can sometimes be utilized successfully, overall therapeutic options for the treatment of candidiasis are limited, and together with the emergence of resistance and the toxicity displayed by some of the presently used antifungal therapies, highlight the urgency in the search for alternative approaches for the therapy of candidiasis (Pierce and Lopez-Ribot, 2013; Pierce et al., 2013, 2015b; Perfect, 2017). We have recently described the identification of a series of small molecule compounds that can serve as the basis for the development of novel anti-virulence strategies for the treatment of *C. albicans* infections (Romo et al., 2017). Our leading candidate, compound 9029936, displays potent inhibitory activity against two of the major virulence traits of *C. albicans*, filamentation and biofilm formation, both *in vitro* and in clinically-relevant murine models of candidiasis (Romo et al., 2017). This anti-virulence mode of action is radically different to that displayed by current antifungals that target fungal growth (Odds et al., 2003). As such, it may represent the basis for effective prophylactic and therapeutic options for the management of candidiasis, including those infections that are recalcitrant to conventional antifungal therapy (Gauwerky et al., 2009). Currently, there are no anti-virulence molecules approved to treat fungal infections although several promising candidates have been reported in the literature targeting *C. albicans* filamentation and biofilm formation, the two main virulence factors utilized for pathogenicity (Shareck and Belhumeur, 2011; Fazly et al., 2013; Pierce et al., 2015a). In the case of bacterial pathogens, anti-virulence approaches are well established as an alternative for antibiotic development and is an area of intensive research (Clatworthy et al., 2007). This further supports the potential therapeutic uses for anti-virulence strategies, which could be added to the alarmingly limited list of antifungal agents.

Here we present a series of experiments with results confirming the true anti-virulence nature of our leading compound and demonstrating its activity against different *C. albicans* clinical isolates, including those displaying multiple underlying mechanisms of antifungal drug resistance, as well as against *C. dubliniensis*. Altogether, our results reaffirm the promise of this leading compound for the development of novel anti-virulence approaches against candidiasis.

## Materials and methods

### Strains and culture conditions

The wild type *C. albicans* strain SC5314 was utilized for the majority of studies. In addition, we used a series of *C. albicans* clinical isolates obtained from HIV-infected patients with oropharyngeal candidiasis (White, 1997a,b), including sets of matched susceptible and resistant isolates for which molecular mechanisms of azole resistance have been previously characterized (White, 1997a,b; Perea et al., 2001); as well as a set of genetically-engineered *C. albicans* “gain of function” strains in key transcriptional regulators of azole resistance (a kind gift from David Rogers) (Schubert et al., 2008; Flowers et al., 2012). We also use representative clinical isolates of several non-*albicans Candida* species, including *C. dubliniensis* isolate 12-1762, *C. tropicalis* isolate 11-2332, and *C. glabrata* isolate 13-1239 (obtained from the Fungus Testing Laboratory at UTHSCSA). For all strains, cell stocks were stored at −80°C and propagated by streaking onto yeast extract peptone dextrose (YPD) agar plates [1% (wt/vol) yeast extract, 2% (wt/vol) peptone, 2% (wt/vol) dextrose, and 1.5% agar], and incubated overnight at 30°C. From these, a loopful of cells was inoculated into flasks (150 ml) containing 25 ml of YPD liquid media in an orbital shaker at 180 r.p.m. and grown overnight for 14–16 h at 30°C. Under these conditions, *Candida* grows as a budding yeast.

### Drugs

Milligram quantities of the lead small molecule compound 9029936 and its structurally related analog 7977044 (with similar activity as described previously) (Romo et al., 2017) were obtained from stock compounds available for hit resupply from Chembridge Corporation (San Diego, CA). A stock solution of fluconazole (Hospira, Lake Forest, IL) prepared in sodium chloride for injection at 2 mg/ml was obtained and stored at 4°C until used. Amphotericin B was obtained in solution at 250 μg/ml (Gibco Life Technologies, Grand Island, NY) and stored at −20°C until used. Caspofungin (Merck & Co., Inc., Whitehouse Station, NJ) was obtained as a powder and was stored at 4°C; a stock solution was prepared in PBS at 2 mg/ml the same day before its addition to well plates.

### Inhibitory effect on *Candida* biofilm formation

These assays used the 96-well flat bottom plate model of *C. albicans* biofilm formation originally developed by our group (Ramage et al., 2001b; Pierce et al., 2008, 2010). For inhibition of biofilm formation, serial 2-fold dilutions (ranging from 20 to 0.039 μM) of investigational compounds were added to the wells of 96-well flat bottom microtiter plates before seeding with aliquots of fungal cells in RPMI-1640 medium supplemented with L-glutamine (Corning, Corning, NY) and buffered with 165 mM morpholinepropanesulfonic acid (Sigma, St. Louis, MO) (100 μL/well of a 1 × 10^6^ cells/ml solution). For *C. dubliniensis*, additional experiments were performed in which biofilms of *C. dubliniensis* clinical isolate 12-1762 were grown in N-Acetyl Glucosamine (GlcNAc) (Shepherd et al., 1980; Hubbard et al., 1985), or Spider media (Liu et al., 1994) using serial 2-fold dilutions ranging from 40 to 0.078 μM. Appropriate positive (absence of drug, to allow for uninterrupted biofilm formation) and negative (no cells, to monitor contamination and to be able to calculate percent inhibition) controls were added. Each compound was assayed at least in duplicate plates (biological replicates), with 4–8 technical replicates per plate. The plates were incubated at 37°C for 24 h to allow for biofilm formation. After the incubation period the plates were washed twice with 200 μL/well of PBS and biofilm inhibition was measured using the 2,3-Bis-(2-Methoxy-4-Nitro-5-Sulfophenyl)-2*H*-Tetrazolium-5-Carboxanilide (XTT) colorimetric assay as previously described by our group (Ramage et al., 2001b; Pierce et al., 2008, 2010). The IC_50_ value, defined as the concentration of each compound leading to 50% inhibition of biofilm formation, was calculated from the results of these assays, using Prism (GraphPad Software Inc., San Diego, CA).

A similar series of experiments was performed in which biofilms were stained with crystal violet to assess biofilm biomass as previously described (O'Toole, 2011). Briefly, after the last incubation, plates were washed twice with PBS and each well was treated with 100 μL of methanol for 20 min for fixation. Methanol was removed and plates allowed to dry. Adherent biofilms were then stained for 10 min with 150 μL of 3% (w/v) crystal violet. After crystal violet was removed and plates were allowed to dry, they were washed thrice with 200 μL of distilled water. For each well, 100 μL of 33% glacial acetic acid was used to dissolve the dye (after microscopy). Glacial acetic acid was left in the wells for 5 min while shaking slowly. Solution was then transferred to a new microtiter plate for OD_550_ measurement to calculate the extent of biofilm inhibition as compared to untreated controls. For microscopy, stained samples were directly observed on the 96-well plate using a 40x objective in an inverted system microscope (Westover Scientific, Mill Creek, WA) equipped for photography. The images were processed for display using Micron software (Westover Scientific).

### Inhibition of filamentation under different growth media conditions

The ability of compound 9029936 to inhibit *C. dubliniensis* filamentation was examined under different growing conditions as previously described (Romo et al., 2017). Media selected include YPD, YPD plus 10% fetal bovine serum (FBS), RPMI 1640, Lee et al. (1975), Spider (Liu et al., 1994), and N-Acetyl Glucosamine (GlcNAc) (Shepherd et al., 1980; Hubbard et al., 1985).

### Invasion assay

For invasion assays, compound 9029936 was added to half of the plate at a concentration of 5 μM. Plates were then placed at 30°C to allow for compound diffusion. A 1:1,000 dilution of *C. dubliniensis* isolate 12-1762 was made from a 2 × 10^6^ cells/ml solution and streaked on to a quadrant with compound 9029936 and on a quadrant without compound. Plates were incubated at 37°C for 5–7 days. Images were taken before and after rinsing plate with water.

### Checkerboard assays for drug combinations of the lead compounds with clinically used antifungal drugs against *C. albicans* biofilms

The effect of the lead compound 9029936 in combination with three commonly used antifungal drugs (fluconazole, amphotericin B, and caspofungin) was determined against planktonic cells, biofilm formation, and preformed biofilms. Briefly, compound 9029936 and antifungals were diluted using a checkerboard method. Rows A-G of the 96 well plate contained serial 2-fold dilutions of the lead compound, ranging from 40 to 0.156 μM, and row H contained no compound. Columns 1-9 of the microtiter plate contained serial 2-fold dilutions of either fluconazole (16–0.25 μg/mL), amphotericin B (8–0.125 μg/mL), or caspofungin (16–0.25 μg/mL); while column 10 contained no antifungal drug. Column 11 of the microtiter plate contained no test compound or drug to serve as a positive control for planktonic growth or biofilm formation and column 12 contained only media to serve as a background control. To assess the effect of the combination therapy on biofilm formation, columns 1–11 of the microtiter plates were seeded with 100 μl of *C. albicans* SC5314 at a concentration of 1 × 10^6^ cells/ml in RPMI containing the appropriate concentrations of test compound and antifungal drugs, and incubated for 24 h at 37°C. Non-adherent cells were removed by washing twice with sterile PBS and inhibition of biofilm formation was determined using the XTT reduction assay. To assess the effect on preformed biofilms, columns 1–11 of microtiter plates were seeded with 100 μl of *C. albicans* SC5314 at a concentration of 1 × 10^6^ cells/ml in RPMI and plates were incubated for 24 h at 37°C to allow for mature biofilms to form. The biofilms were washed twice with sterile PBS and 100 μl fresh RPMI media containing the appropriate dilutions of test compound and antifungal drug were added to the wells and biofilms were incubated for an additional 24 h prior to the final washing with sterile PBS and quantification using the XTT reduction assay. Percent biofilm inhibition was determined for each combination of compound and drug. To assess the effect of the combination therapy on planktonic growth, columns 1–11 of the round bottom microtiter plates were seeded with 200 μl of *C. albicans* SC5314 at a concentration of 0.5 × 10^3^ cells/ml in RPMI-1640 containing the appropriate concentrations of test compound and antifungal drugs then incubated for 24 h at 30°C. Effect on planktonic growth was determined using the OD_600_ readings.

In all cases, using the calculated minimum inhibitory concentration for each compound and antifungal alone and the combination of the compound with antifungal, the fractional inhibitory concentration (FIC) for the pair was used to determine and used to detect if synergism, indifference, or antagonism was present when biofilms were treated with a combination of compounds and antifungal drugs. The value of the ∑FIC index is used to interpret the nature of the interactions: synergism ≤ 0.5, indifference >0.5 to ≤ 4, antagonism >4 (Hindler, 1983, 1994, 1995; Jorgensen et al., 1994).

### Serial passage experiments for induction of resistance

Two individual populations of *C. albicans* were founded from a single colony of *C. albicans* SC5314. One culture was grown in YPD medium at 37°C in the absence of serum (non-filamenting inducing conditions), the other was grown in RPMI-1640 at 37°C (filament-inducing conditions). For each condition, cultures were grown without any treatment (as control) or in the presence of 5 μM of 9029936 compound, a concentration previously determined to inhibit biofilm formation and filamentation without affecting cell growth (Romo et al., 2017). Cultures were placed in orbital shakers and every day 10 μl from each culture were serially transferred into 3 ml of the corresponding fresh medium, YPD or RPMI-1640 in the presence or absence of 5 μM of the leading compound. These transfers continued daily for 56 days, maintaining the concentration of 9029936 at 5 μM. After daily transfers, population samples were stored in 1 ml of 40% (vol/vol) glycerol at −80°C. To assess for the development of resistance, cultures grown in the presence of the small molecule compound were monitored for the presence/absence of hyphae before daily transfers microscopically (not shown). Cells from the stored populations were streaked, grown overnight, and used to seed the wells of 96-well microtiter plates in the presence of compound 9029936 at concentrations ranging from 40 to 0.078 μM in RPMI and incubated at 37°C for 24 h to assess inhibitory activity against biofilm formation. For filamentation studies, cells from stored populations were streaked, grown overnight, and used to seed the wells of 96-well microtiter round bottom plates in the presence of compound 9029936 at concentrations ranging from 40 to 0.078 μM in YPD plus 10% FBS and incubated at 37°C for 6 h to assess the activity of compound 9029936 against filamentation. Microscopy was performed using differential interference contrast (DIC) on an Axio Observer D1 inverted microscope (Carl Zeiss, Thornwood, NY) equipped for photography.

### Cell viability staining

The effect of compound 9029936 on *C. albicans* and *C. dubliniensis* cell viability was assessed using the viability staining protocol and kit (Invitrogen, Carlsbad, CA). Briefly, fungal cells were grown in the presence of compound 9029936 at varying concentrations (2.5, 5, 10, 20, and 40 μM) in RPMI and incubated at 37°C for 24 h while shaking. After incubation cells were washed twice with PBS and a 1:1,000 dilution was stained with 10 μM of FUN-1 for 1 h at 30°C in the dark. Cells were then spun at 5,000 rpm for 5 min and FUN-1 was removed. Cells were then re-suspended in 40 μL of Calcofluor white (CFW) at a concentration of 25 μM for 10 min at room temperature. Fluorescent and DIC microscopy was performed using an Axio Observer D1 inverted microscope (Carl Zeiss) equipped for photography.

### Examination of fungal cellular states using imaging flow cytometry

A 1:30 dilution of an overnight culture of *C. albicans* SC5314 was used to inoculate a 96-well round bottom microtiter plate containing compound 9029936 at a concentration of 10 μM in 100 μL of RPMI-1640 medium and incubated for 6 h at 37°C with 5% CO_2_. After incubation, cells were washed twice with PBS, followed by incubation with a solution containing 2.5 μg/ml FM4-64 FX membrane staining dye (Thermo-Fisher Scientific, Waltham, MA) and 0.4 μg/ml Calcofluor white (CFW) in a modified phosphate buffer saline, pH 8.0 for 30 min at 37°C. Following staining, wells were washed and re-suspended in 30 μL of 2% paraformaldehyde solution. Samples were analyzed with an ImageStreamX MKII (Millipore, Burlington, MA) image flow cytometer with a 7-μm core at low flow rate and high sensitivity using INSPIRE software. Fluorochromes were excited with 405, 561, and 785 nm lasers and image data was collected through a 60 × objective in appropriate channels. Single-color reference samples for each fluorochrome were generated by inclusion of cells that are incubated with each fluorochrome separately. Images were analyzed using IDEAS® software version 6.2 (Millipore, Burlington, MA). A compensation matrix was built with the data from single-color reference samples to allow removal of spectral spillover to adjacent channels from each detection channel. Single-cell images were first gated on the focused cells with a size (area) range between 50 and 200 pixels (~15–60 μm^2^). A dot plot of bright field area vs. aspect ratio (width/length) was used to distinguish yeast, elongated cells, and hyphae subpopulations that have aspect ratios (*r*) ranging from *r* >0.75, 0.75≥ *r* ≥0.38, and *r* < 0.38, respectively. Cellular states including cell length, CFW, and FM4-64 binding indexes were measured and plotted. To accommodate the total CFW and FM4-64, intensity increased when cell size enlarged during yeast-hypha transition, the binding indexes were defined as the ratios of the sum of the pixel intensities of CFW and FM4-64 in the cell masks divided by the cell length (intensity/length).

### Hydroxyurea genotoxic stress assay

Hydroxyurea assays for genotoxic stress were performed as previously described (Fazly et al., 2013). Briefly, *C. albicans* SC5314 cells from an overnight culture were washed twice with PBS and a 1:30 dilution was used to seed a 96-well round bottom microtiter plate containing compound 9029936 (ranging from 40 to 0.078 μM) as well as hydroxyurea (HU) at a concentration of 100 μM (concentration shown to induce filamentation). Positive (cells in the presence of compound 9029936 only) and negative (cells in the presence of 100 μM hydroxyurea only) controls were included. Plates were incubated at 37°C for 24 h. Microscopy was performed using DIC on an Axio Observer D1 inverted microscope (Carl Zeiss) equipped for photography.

### Eurofin cerep safetyscreen 44 panel for *in vitro* pharmacological profiling and detection of off-target effects

For pharmacological profiling and determination of off target effects, the leading 9029936 compound was screened at a 10 μM concentration in duplicate for potential to bind to a broad panel of receptors, enzymes, and ion channels in a commercial screen (SafetyScreen 44, Eurofins Cerep-Panlabs, France). Details and experimental conditions for these assays can be found online at www.cerep.fr. For a list of specific assays please refer to **Figure 8**.

## Results

### Activity of compound 9029936 against azole resistant clinical isolates and gain-of-function strains

We performed susceptibility testing of compound 9029936 against a number of clinical isolates recovered form HIV-infected patients with oropharyngeal candidiasis, including several isolates that are resistant to treatment with conventional antifungals (White, 1997a,b). These susceptibility assays were conducted for biofilm inhibition. In addition, we also examined the activity of our leading compound against several gain-of-function strains, which are laboratory generated strains overexpressing key regulators of the ergosterol pathway and drug resistance efflux pumps (Schubert et al., 2008; Flowers et al., 2012); more specifically these included a gain-of-function strain in *UPC2*, leading to overexpression of *ERG11*, the gene encoding the target enzyme of azole antifungals, as well as gain-of-function strains in *MRR1* and *TAC1*, leading to overexpression of *MDR* and *CDR* genes respectively, encoding a major facilitator and an ABC transporter involved in antifungal drug resistance (Schubert et al., 2008; Flowers et al., 2012). Table 1 contains information on each of these strains as well as the IC_50_ values obtained from the biofilm inhibition assays. As in Table 1, the IC_50_ values obtained using each of the strains and isolates were mostly comparable (although slightly elevated for strains Sc*MRR1*R34A, Sc*TAC1*R34A, 2440, and 3731) to those values determined against the laboratory strain SC5314, therefore validating the activity of compound 9029936 against multiple *C. albicans* strains with decreased susceptibility against conventional antifungals, including both clinical isolates and laboratory-generated strains.

**Table 1:** Biofilm inhibitory activity of compound 9029936 against *C. albicans* clinical isolates and gain-of-function strains.

**Strain/Isolate**	**Description**	**Overexpressed genes**	**IC_50_ (biofilm inhibition) in μM**
SC5314	Wild-type laboratory strain	N/A	1.875
**CLINICAL ISOLATES FROM AIDS PATIENT (DEVELOPED RESISTANCE OVER 2 YEARS OF FLUCONAZOLE TREATMENT)**			
TW1	Clinical isolate (matched susceptible isolate for this series)	N/A	0.8022
TW2	Clinical isolate (drug resistance observed)	*MDR1*	0.6031
TW3	Clinical isolate (drug resistance observed)	*MDR1*	2.638
TW17	Clinical isolate (multi drug resistance observed) Point mutation: R467K	*CDR1, MDR1, ERG11*	2.767
**GAIN-OF-FUNCTION STRAINS OVEREXPRESSING REGULATORS OF AZOLE RESISTANCE**			
SCR (GOF Control)	Wild-type strain background used to generate gain-of-function strains	N/A	1.814
Sc*UPC2*R14A	Strain with homozygous activating mutation in *UPC2* (G648D) (*UPC2*^G648D^–FRT/*UPC2*^G648D^–FRT	*UPC2* and *ERG11*	1.569
Sc*MRR1*R34A	Strain with homozygous activating mutation in *MRR1* (P683S) (*MRR1*^P683S^–FRT/*MRR1*^P683S^–FRT	*MRR1* and *MDR1*	5.494
Sc*TAC1*R34A	Strain with homozygous activating mutation in *TAC1* (6980E) (*TAC1*^6980E^–FRT/*TAC1*^6980E^–FRT	*TAC1, CDR1* and *CDR2*	4.400
**FLUCONAZOLE RESISTANT CLINICAL ISOLATES**			
4639	F449S, T229A (Erg11p substitutions)	*MDR1, CDR1*	1.424
4617	F449S, T229A (Erg11p substitutions)	N/A	0.9198
6482	D116E, K128T, Y132H, D278N, G464S, P230L (Erg11p substitutions)	N/A	3.011
2440	V437I (Erg11p substitution)	*MDR1, ERG11*	5.645
3731	F126L, K143R (Erg11p substitutions)	*MDR1*	5.069
412	K128T (Erg11p substitution)	N/A	1.474

*The IC_50_ values were determined from dose-response experiments looking at the ability of the compound to inhibit biofilm formation by the different C. albicans strains*.

### Activity of compound 9029936 and 7977044 against non-*albicans Candida* species

**Figure 1:**
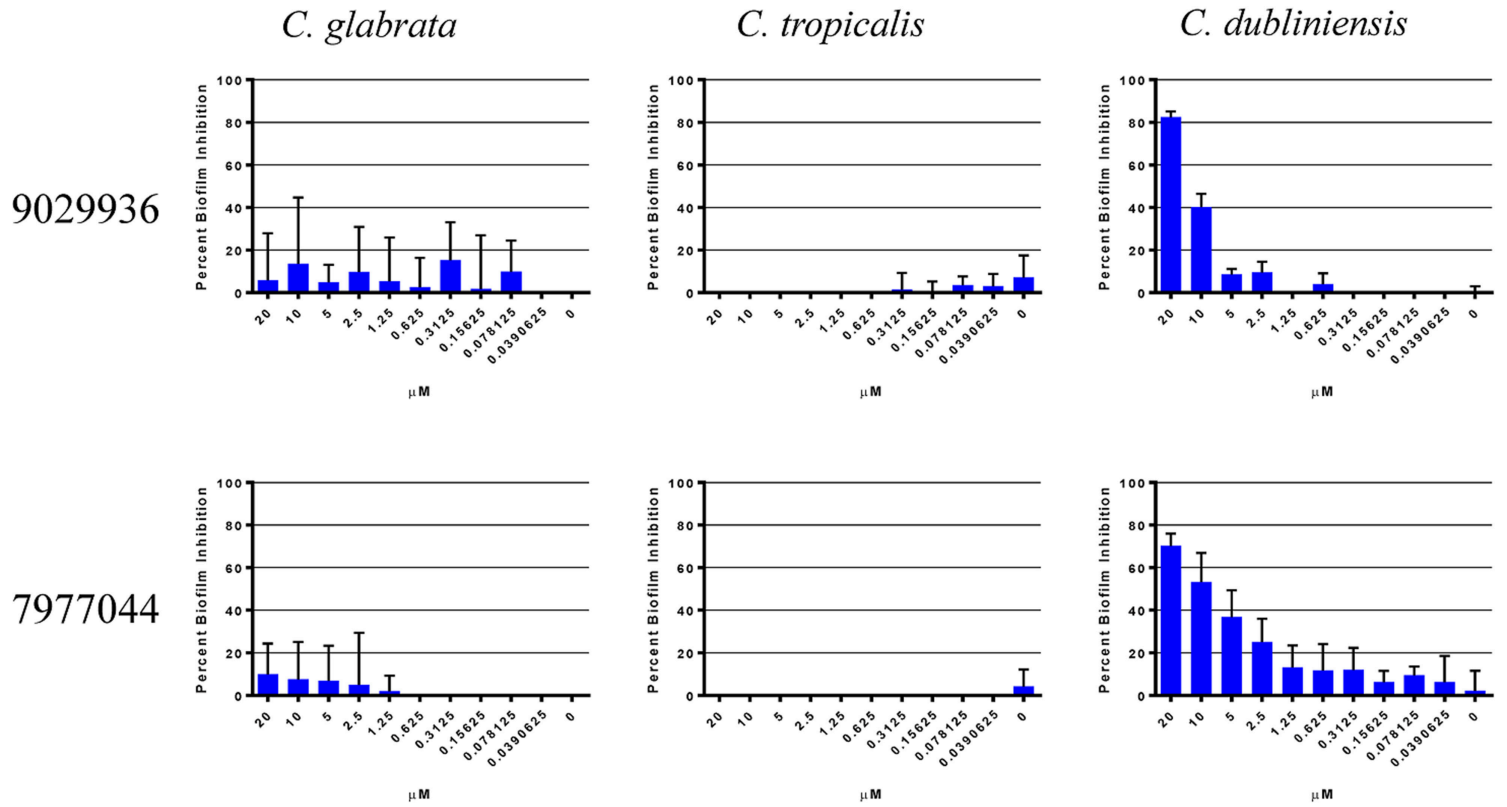
Activity of compound 9029936 against biofilm formation by non-*albicans Candida* species. *C. glabrata, C. tropicalis, and C. dubliniensis* cells were grown in RPMI 1640 to assess biofilm formation. The compounds were tested in serial 2-fold dilutions with concentrations ranging from 20 to 0.039 μM, with appropriate positive and negative controls. Results are shown as mean percent biofilm inhibition compared to control biofilms (in the absence of compound) as determined by the XTT colorimetric assay for multiple technical replicates from several independent experiments, error bars indicate standard deviations.

We have previously described that compounds 9029936 and its analog 7977044 were able to inhibit biofilm formation in *C. albicans* (Romo et al., 2017), most likely as a direct consequence of their inhibitory effects on filamentation, since in *C. albicans* these two processes are inextricably linked (Ramage et al., 2001c, 2005; Lopez-Ribot, 2005; Pierce et al., 2014). A true anti-virulence agent should, in theory, be highly specific to the organism or species that employs that virulence factor (Clatworthy et al., 2007; Rasko and Sperandio, 2010; Pierce et al., 2015a). Not all *Candida* species are capable of undergoing the yeast to hyphae transition, and one would expect that our leading compounds may display different levels of activity against different *Candida* species depending on their intrinsic ability to filament. Moreover, different *Candida* spp. also display varying abilities to form biofilms (Gilfillan et al., 1998; Ramage et al., 2001a; Jabra-Rizk et al., 2004; Silva et al., 2012; Brunke and Hube, 2013; Sardi et al., 2013), which may or not be related to their ability to filament. Thus, in an initial set of experiments we examined the biofilm inhibitory activity of the two leading compounds from this series against other non-*albicans Candida* species, the species selected for these experiments were ones known to form biofilms associated with filamentation (*C. tropicalis* and *C. dubliniensis*), as well as *C. glabrata*, which is capable of forming biofilms independent of filamentation (Silva et al., 2012; Brunke and Hube, 2013; d'enfert and Janbon, 2016). It is important to note that these non-*albicans* species in general are unable to form robust and durable biofilms like those of *C. albicans*. As shown in Figure 1, the only species against which the compounds displayed biofilm-inhibitory activity was *C. dubliniensis*. These results are not surprising, since *C. dubliniensis* is the closest related organism to *C. albicans* (Gilfillan et al., 1998; Sullivan and Coleman, 1998). Both *C. glabrata* and *C. tropicalis* were able to form biofilms uninhibited by the presence of the compounds. In *C. glabrata*, this is probably due to the inability to filament, which further underscores the specificity of the compounds and their anti-virulence nature. In the case of *C. tropicalis*, although presence of filamentous forms is observed in biofilms formed by this species, in general the extent of filamentation is much lower than in the case of *C. albicans*, and even *C. dubliniensis* (Silva et al., 2012; Lackey et al., 2013; Zhang et al., 2016), and thereby biofilm formation in this species overall may not depend solely on its ability to filament. Based on these results, we decided to study the activity of our lead compound 9029936 against *C. dubliniensis* filamentation and biofilm formation in more detail. Similar to its close relative *C. albicans*, these represent the two main virulence factors that are inherently linked to *C. dubliniensis* pathogenesis (Ramage et al., 2001a).

### Effects of compound 9029936 on *C. dubliniensis*: inhibition of agar invasion, filamentation, and biofilm formation under multiple environmental conditions

**Figure 2:**
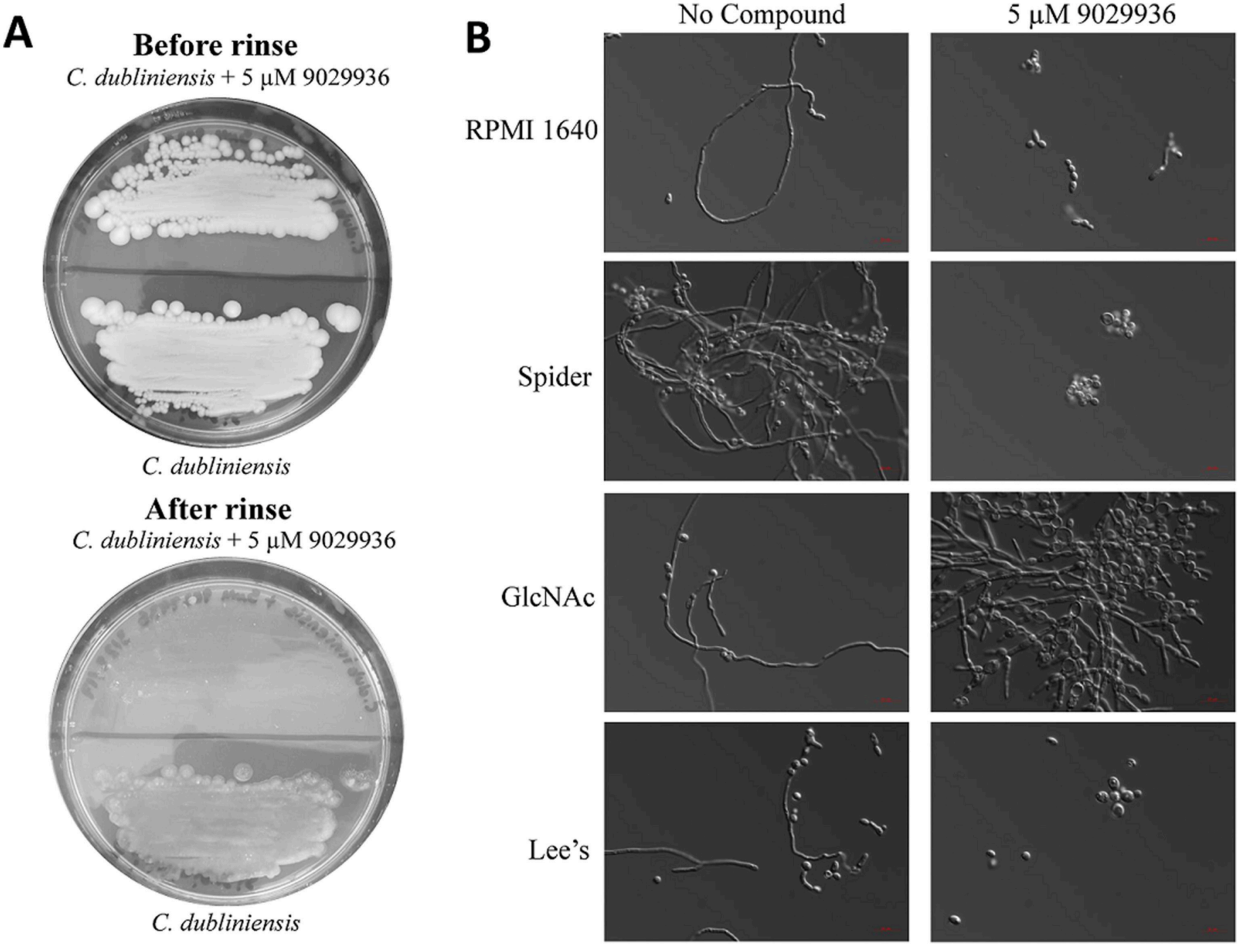
Activity of compound 9029936 against *C. dubliniensis* in an agar invasion assay and filament stimulating conditions. **(A)** Compound 9029936 inhibits *C. dubliniensis* agar invasion. *C. dubliniensis* isolate 12-1762 cells were streaked on YPD plates with or without compound 9029936 at 5 μM and the plates incubated at 37°C for 5 days. The plates were photographed prior to (top panel) and after (bottom panel) surface cells were removed by gentle washing under running water. In the presence of the compound cells grew mostly on the surface of the medium and were washed away, whereas cells grown in the absence of the compound had invaded the agar and remained after washing. **(B)** Inhibition of filamentation in different liquid media. Liquid cultures of *C. dubliniensis* isolate 12-1762 were grown in a variety of hypha-inducing media in the absence or presence of compound 9029936 at a concentration of 5 μM. Aliquots were taken from the different cultures at 6 h post-induction, visualized by DIC microscopy and photographed. Bars are 20 μm in all panels.

As shown previously, compound 9029936 displayed potent filamentation inhibition against *C. albicans* (Romo et al., 2017). We also concluded that the compound displayed activity against *C. dubliniensis* in the biofilm inhibition assay (Figure 1). Therefore, we hypothesized that the similar activity seen against *C. dubliniensis* is due to its close relatedness to *C. albicans* (Gilfillan et al., 1998; Sullivan and Coleman, 1998; Sullivan et al., 2005; McManus et al., 2008; Citiulo et al., 2009; Spiering et al., 2010). To further characterize this activity, we conducted an agar invasion assay as previously described (Romo et al., 2017). Briefly, *C. dubliniensis* cells from an overnight culture were streaked on to a YPD agar plate lacking or containing compound 9029936 at 5 μM concentrations and incubated at 37°C for 5–7 days. As expected, the untreated *C. dubliniensis* invaded the agar, while compound 9029936 inhibited filamentation and agar invasion (Figure 2A). Overall, these results indicate that similarly to the activity displayed against *C. albicans*, compound 9029936 strongly inhibited filamentation and agar invasion by *C. dubliniensis*.

To further characterize the filamentation inhibitory activity of compound 9029936 against *C. dubliniensis*, cells were grown under strong filament inducing conditions in the presence or absence of compound 9029936 at 5 μM concentrations and incubated at 37°C for 48 h. The conditions tested included YPD, YPD plus 10% FBS, Lee's medium (Lee et al., 1975), RPMI 1640, GlcNAc (Shepherd et al., 1980; Hubbard et al., 1985), and Spider medium (Liu et al., 1994). *C. dubliniensis* is unable to filament as effectively as *C. albicans* (Moran et al., 2007; Stokes et al., 2007; Spiering et al., 2010), and therefore, induction of filamentation through multiple media conditions was required to identify a suitable environment in which to study its filamentation. As show in Figure 2B, *C. dubliniensis* was able to filament under all four conditions tested, while the presence of compound 9029936 blocked this morphological transition. We note that in GlcNAc media, *C. dubliniensis* did not form true hyphae, but instead was able to undergo the morphological transition into pseudohyphal forms.

**Figure 3:**
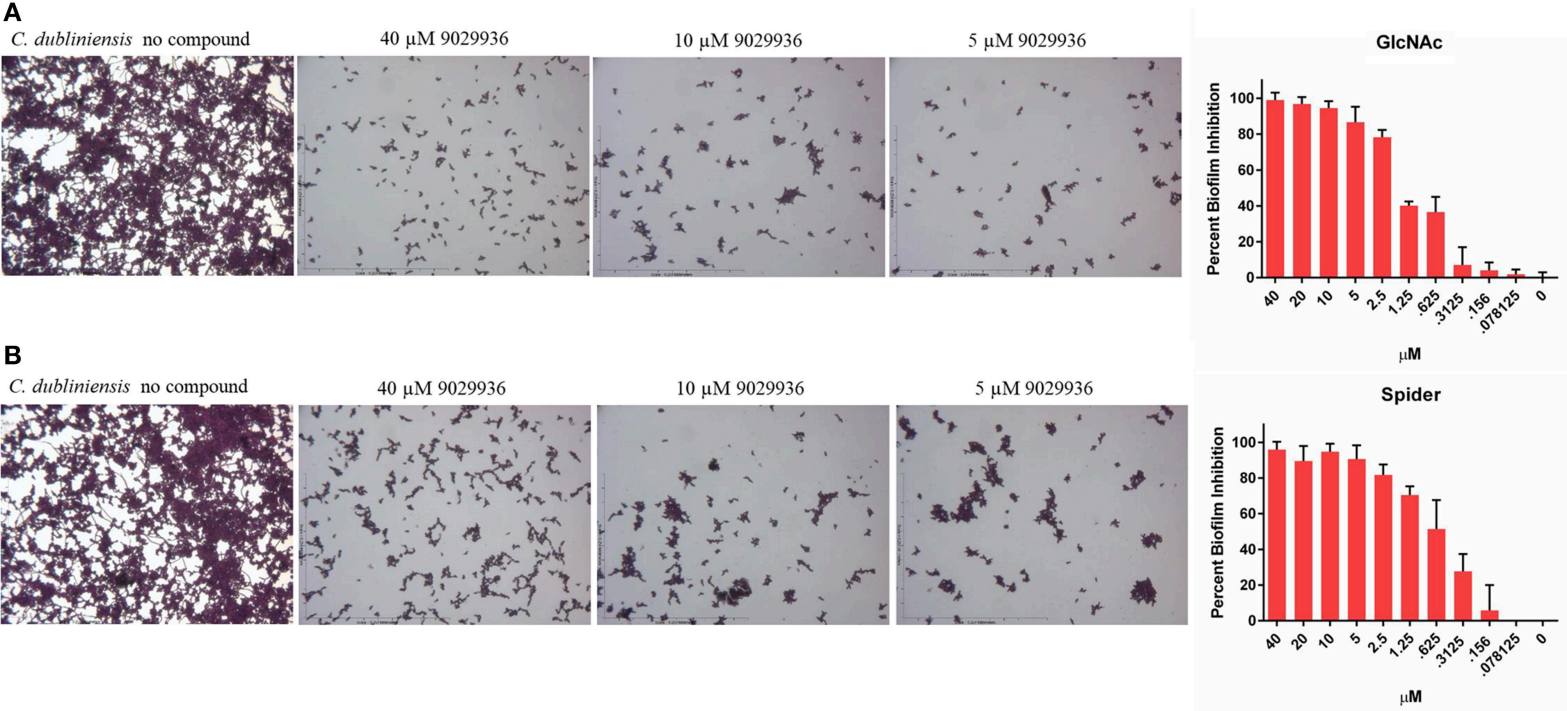
Activity of compound 9029936 against biofilm formation by *C. dubliniensis. C. dubliniensis* isolate 12-1762 cells were grown in **(A)** GlcNAc or **(B)** Spider media to assess biofilm formation. The compound was tested in serial 2-fold dilutions with concentrations ranging from 40 to 0.078 μM, with appropriate positive and negative controls. Results are shown as mean percent biofilm inhibition compared to control biofilms (in the absence of compound) as determined by the Crystal violet biomass assay for multiple technical replicates from several independent experiments, error bars indicate standard deviations.

Since compound 9029936 was able to inhibit agar invasion (Figure 2A) as well as filamentation under several inducing conditions (Figure 2B), we posited that it would also be able to inhibit biofilm formation by *C. dubliniensis* in different growth media, besides the most common RPMI used in our initial experiments. Thus, *C. dubliniensis* biofilms were grown in 96-well microtiter plates using either Spider (Shepherd et al., 1980; Hubbard et al., 1985; Liu et al., 1994) or GlcNAc (Shepherd et al., 1980; Hubbard et al., 1985) media. As seen in Figure 3, these conditions led to robust biofilm formation. However, the presence of compound 9029936 at concentrations as low as 5 μM was able to prevent biofilm formation by this *C. dubliniensis* strain. From these experiments, the calculated IC_50_ values were 0.5582 and 1.264 μM for both Spider and GlcNAc media, respectively, which are similar to those observed for *C. albicans* in regular RPMI medium.

### Drug combination assays with compound 9029936 and currently used antifungal agents

Although drug combinatorial therapies are common when treating viral and bacterial infections, they have not been thoroughly implemented in the clinic for the treatment of fungal infections (Baddley and Pappas, 2005; Spitzer et al., 2017). The anti-virulence nature of compound 9029936 makes it an ideal candidate for combinatorial therapy with currently used antifungals. In order to investigate the activity of combinations between our leading compound and current antifungals we performed checkerboard assays using the planktonic, biofilm inhibition, and pre-formed biofilm assays (Table 2). These assays allow for the calculation of the fractional inhibitory concentration index (FICI), which allows for the identification of synergistic, indifferent, or antagonistic interactions between the compounds in question (Hall et al., 1983; Hindler, 1983, 1994; Jorgensen et al., 1994). A FICI of 0.5 or less indicates synergy, while numbers >0.5 indicate indifference. Any number equal to or higher than 4 is indicative of antagonistic interactions (Hall et al., 1983; Hindler, 1983, 1994; Jorgensen et al., 1994). The antifungals used in these experiments were fluconazole, caspofungin, and amphotericin B, representatives of each of the three main classes of clinically-used antifungals, the azoles, the echinocandins, and the polyenes (Odds et al., 2003). Although we only observed synergistic activity for the combination of compound 9029936 and caspofungin against pre-formed biofilms, we observed indifference for all other combinations and conditions tested. In no instance we observed antagonism, suggesting that compound 9029936 could be potentially used in combination with currently used antifungal drugs. Similar results were obtained for compound 7977044 (data not shown).

**Table 2:** Fractional inhibitory concentration indices from drug combination assays.

**Drug combinations**	**Biofilm inhibition**	**Pre-formed biofilms**	**Planktonic**
9029936 + Fluconazole	Indifference (FICI = 1.8)	Indifference (FICI = 1.625)	Indifference (FICI = 1.187)
9029936 + Amphotericin B	Indifference (FICI = 1.06)	Indifference (FICI = 1.02)	Indifference (FICI = .6875)
9029936 + Caspofungin	Indifference (FICI = 1.3125)	Synergy (FICI = .4082)	Indifference (FICI = 1.328)

*Compound 9029936 was assessed in combination with conventional antifungal drugs to determine inhibitory effects on C. albicans strain SC5314 planktonic growth, biofilm formation, and against preformed biofilms*.

### Serial passage experiments in the presence of the lead compound to assess the potential for the development of resistance

**Figure 4:**
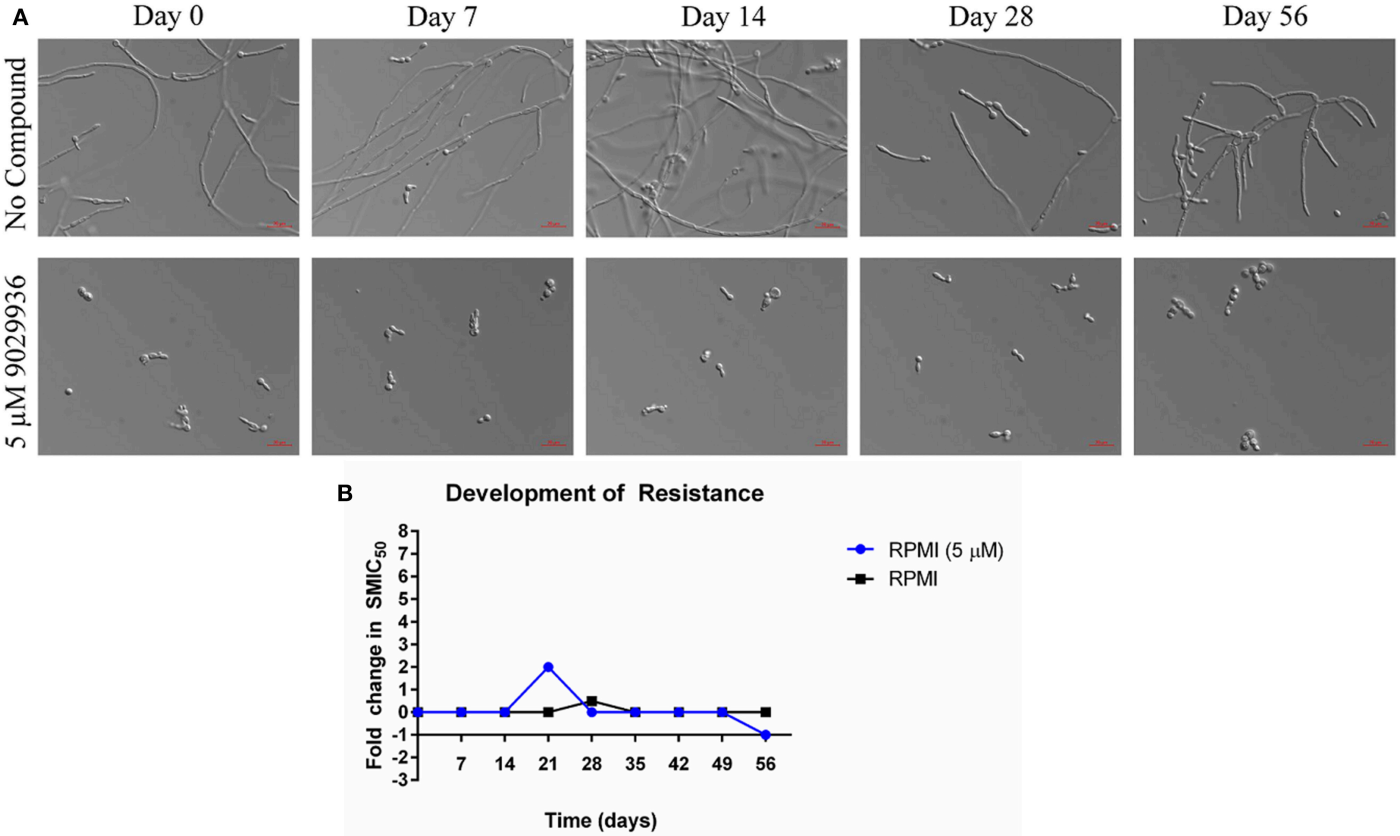
Serial passage of *C. albicans* SC5314 in the presence of 9029936. *C. albicans* SC5314 cells were grown in the absence or presence of compound 9029936 at a concentration of 5 μM and passaged in this concentration for 56 days in order to investigate the development of resistance. **(A)** Ability of the lead compound to still inhibit filamentation in isolates recovered after the number of serial passages indicated both in the presence and absence of compound 9029936. **(B)** The ability of the small molecule compound to still inhibit biofilm formation was determined using the same microtiter plate model methodology, with isolates recovered after the number of serial passages indicated both in the presence and absence of compound 9029936.

As shown previously, compound 9029936 behaves as a true anti-virulence agent by inhibiting *C. albicans* filamentation without having any significant effect on growth (Romo et al., 2017). Indeed, the reason microorganisms develop resistance to a specific drug is because most drugs target components or processes vital for cell growth and survival (Blair et al., 2015; Hughes and Andersson, 2015; Fairlamb et al., 2016). It is this selective pressure that forces the microorganism to adapt and survive (Blair et al., 2015; Hughes and Andersson, 2015; Fairlamb et al., 2016). A true anti-virulence agent should in theory not induce (or should be able to delay) the development of resistance because it targets virulence factors (Clatworthy et al., 2007; Rasko and Sperandio, 2010; Ling et al., 2015), which in opportunistic pathogens such as *C. albicans*, are not necessary for cell growth and survival. In order to further validate compound 9029936 as a true anti-virulence agent, we performed assays to attempt to induce the development of resistance in *C. albicans* as previously described (Pierce et al., 2015a). Briefly, *C. albicans* was grown under filament inducing conditions in the presence or absence of 5 μM of compound 9029936 and passaged every day into fresh media containing the compound at the same concentration for 8 weeks. Each day, an aliquot was collected and frozen for future studies. During this process samples were also checked microscopically to assess filamentation (untreated samples) or the lack of filamentation (treated samples). No filamentation was seen in the treated samples through the duration of these experiments. To test if resistance was developed to compound 9029936, each isolate was streaked, grown overnight, and used in filamentation assays (Figure 4A) as well as in biofilm formation assays (Figure 4B). All isolates were able to filament properly under filament inducing conditions when compound 9029936 was absent. In comparison, compound 9029936 at 5 μM was able to inhibit filamentation under filament inducing conditions by all of the isolates, indicating that no resistance to the compound was developed under the conditions tested, even after several weeks of serial passages. Moreover, the isolates were tested using the biofilm inhibition assay to assess fold changes in SMIC_50_ (sessile minimum inhibitory concentration) (Figure 4B). As expected, all isolates displayed an SMIC_50_ comparable to that of day 0, with only a slight transitory increase detected for the isolate recovered on day 21. Overall, these results indicate that repeated exposure to the lead compound is highly unlikely to foster the emergence of resistance.

### The lead compound is not toxic to *Candida* cells at concentrations higher than those needed to inhibit filamentation

**Figure 5:**
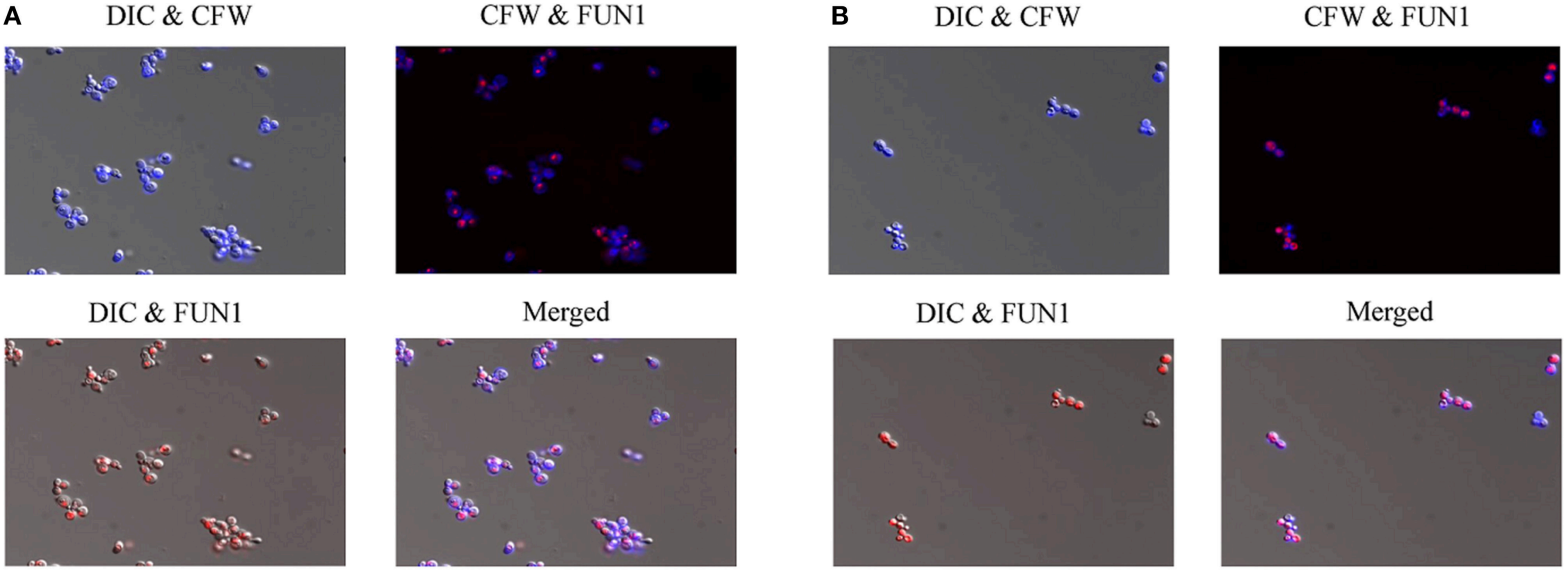
Cell viability staining of *C. albicans* and *C. dubliniensis* in the presence of compound 9029936. **(A)** *C. albicans* SC5314 and **(B)** *C. dubliniensis* isolate 12-1762 cells were grown at 37°C in the presence of compound 9029936 at 40 μM for 24 h. Cells were then stained with FUN1 and Calcoflour white (CFW) to assess live and dead cell populations. Samples were visualized by fluorescent and DIC microscopy and photographed.

In order to further investigate the effects of compound 9029936 on cell growth and confirm its true anti-virulence activity, we performed viability staining of *C. albicans* SC5314 and *C. dubliniensis* isolate 12-1762 cells grown in the presence of different concentrations of the leading compound. Briefly, *C. albicans* SC5314 and *C. dubliniensis* isolate 12-1762 cells were grown at 37°C in the presence of compound 9029936 at concentrations ranging from 2.5 to 40 μM for 24 h. After incubation, cells were washed with PBS and stained with FUN1 (indicator of cell viability), followed by Calcofluor white (stains chitin in fungal cell walls), and visualized using a fluorescence microscope. If cells are viable, they will uptake FUN1 and the dye will concentrate in vesicles (red), while calcofluor white should stain all cells (blue). Figure 5 shows the images from the 40 μM concentration samples of *C. albicans* SC5314 (Figure 5A) and *C. dubliniensis* isolate 12-1762 (Figure 5B). Another set of experiments was conducted at 30°C, which yielded similar results (not shown). As seen in the Figure 5, compound 9029936 did not compromise cell viability even at 40 μM, the highest concentration tested, which is consistent with previous growth curve observations previously described by our group (Romo et al., 2017). Although fungistatic activity was observed at concentrations higher than 40 μM (which are not physiologically relevant), no fungicidal activity was observed during our studies.

### The drug-treated *C. albicans* cells display changes on cell size, shape, and integrity of cell wall and membranous system

**Figure 6:**
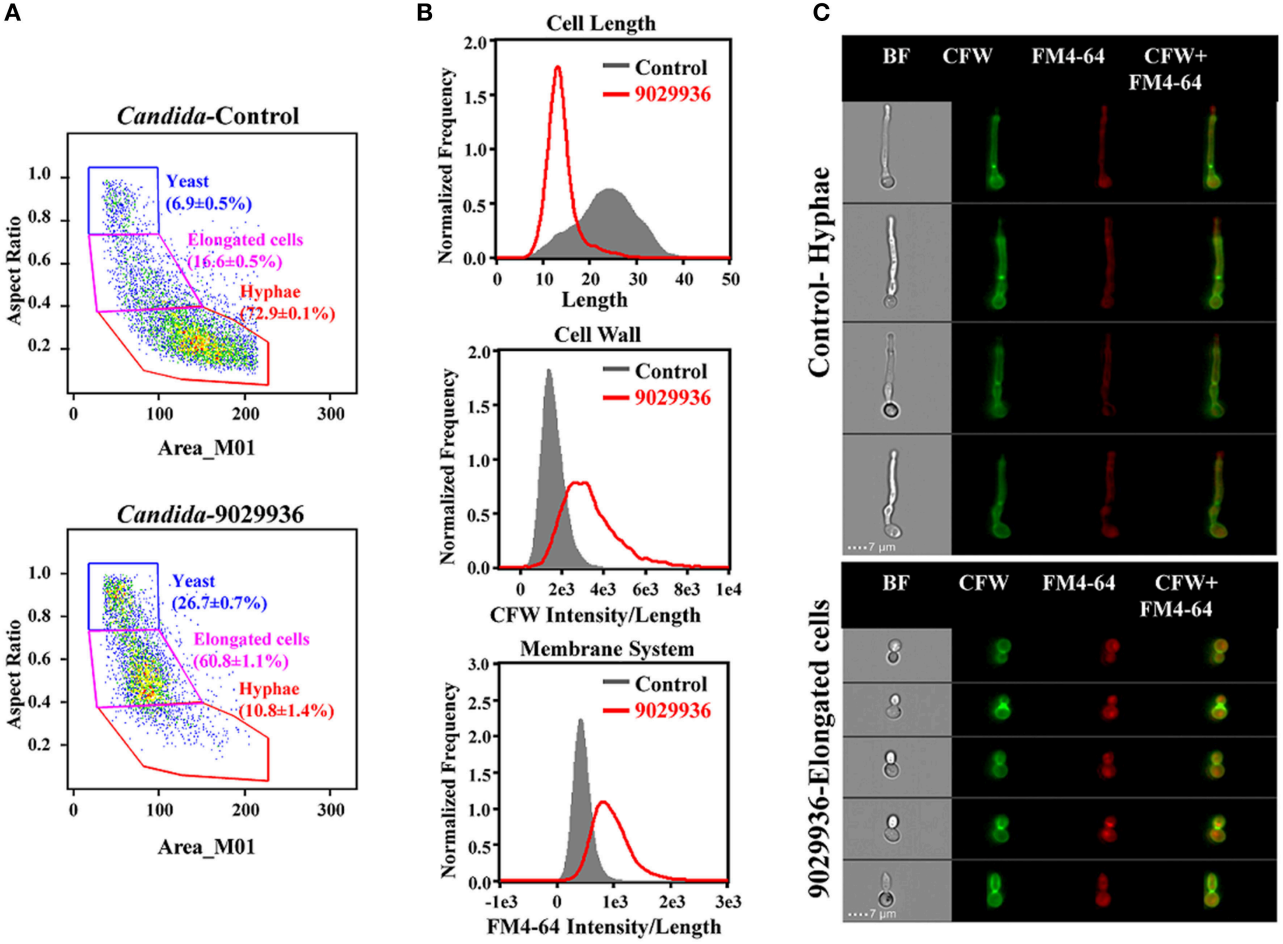
Quantitative measurement of cellular states after treatment with compound 9029936 using image flow cytometry. *C. albicans* SC5314 cells were grown in the presence of compound 9029936 at a concentration of 10 μM for 6 h compared to untreated control cells. Samples were then washed and stained with Calcofluor white (CFW) for cell wall and FM4-64 for hydrophobic membranes. **(A)** Cells were classified into 3 subpopulations based on their properties of area vs. aspect ratio. Results showed that the treated cells grew into elongated cells while majority of untreated yeast grew into hyphae (*P* < 0.0001; Student *T*-test). **(B)** Comparison of cellular states including cell length, CFW, and FM4-64 binding indexes for assessment of cell wall and membrane integrity between treated (red) and untreated control (gray) samples. **(C)** Representative images of control and treated cells showed drug effects on cell size, shape and integrity of cell wall and membrane system. Samples were acquired on an Amnis ImageStream Mark II imaging flow cytometer and analyzed using IDEAS software.

In order to characterize the physiological effects of treatment with compound 9029936 on *C. albicans* cells, we utilized imaging flow cytometry to quantitatively measure cellular states. This technology combines the speed, sensitivity and phenotyping abilities of flow cytometry with the detailed imaging and functional insights of confocal microscopy, which allows for the visualization of effects on individual cells. Both control and treated cells were labeled with CFW and FM4-64 to assess the effects of the drug treatment on cell morphology, cell wall, and membrane integrity (Figure 6). As seen in Figure 6A, and consistent with its inhibitory effects on filamentation, *C. albicans* cells treated with compound 9029936 at 10 μM for 6 h showed a much less elongated morphology as compared to untreated cells (Figures 6A–C). While the majority of control cells grew into hyphae (72.9 ± 0.1%; 23.5 ± 6.1 in length), the treated cells only grew into elongated cells (60.87 ± 1.1%; 13.7 ± 3.3 in length). The treated cells also had compromised cell walls and vacuolar membrane systems, indicated by a significant increase in CFW and FM4-64 binding indexes (gray vs. red) in Figure 6B. Representative images of hyphae from the control samples and elongated cells from the treated *Candida* cells are shown in Figure 6C. These results point to the possibility that compound 9029936 could affect proper cell wall and vacuolar membrane assembly. This could be partly due to the secondary effects of the filament inhibitory properties, which might be preventing the proper assembly or incorporation of cell wall and vacuole components.

### Compound 9029936 inhibits filamentation induced through the genotoxic stress pathway

**Figure 7:**
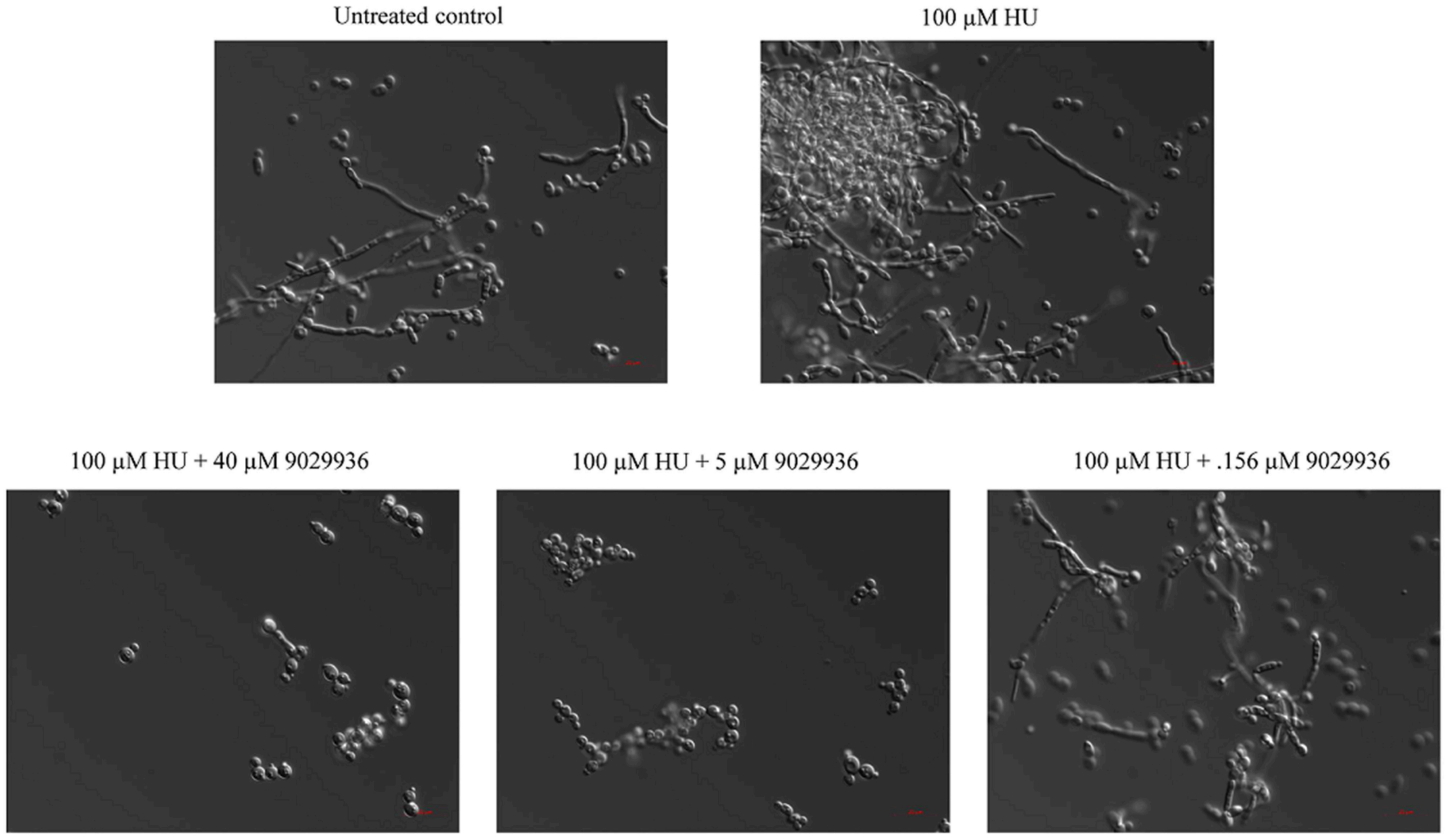
Inhibition of filamentation through genotoxic stress. *C. albicans* SC5314 cells were grown under non-filament inducing conditions (YPD at 30°C) in the presence or absence of hydroxyurea (HU) at a concentration of 100 μM and compound 9029936 at concentrations ranging from 2.5 to 40 μM for 24 h. Samples were visualized by DIC microscopy and photographed.

Compound 9029936 was shown previously to inhibit *C. albicans* filamentation induced through all different environmental conditions tested (Romo et al., 2017). Most recently genotoxic stress has also been demonstrated to induce *C. albicans* filamentation through a damage response pathway (Fazly et al., 2013). In order to investigate the activity of compound 9029936 against filamentation induced through genotoxic stress, *C. albicans* was exposed to hydroxyurea at a concentration of 100 μM for 24 h at 30°C (non-filament inducing conditions) in the presence or absence of compound 9029936 at concentrations ranging from 0.078 to 40 μM (Figure 7). Hydroxyurea induced filamentation (through genotoxic stress) when it was present at 100 μM in the absence of compound 9029936. In contrast, compound 9029936 was able to inhibit filamentation induced through this pathway at concentrations as low as 5 μM (Figure 7). These results indicate that compound 9029936 prevented filamentation through less conventional pathways such as genotoxic stress, and extend the number of environmental conditions, impinging upon different regulatory pathways, under which it is capable to exert potent inhibitory activity of this key *C. albicans* morphogentic conversion intimately linked to virulence. Although these results do not point to an exact mechanism of action of the lead compound, they are relevant to the challenging environment encountered by *C. albicans* inside the human host, which can cause stress and damage to the fungus possibly leading to filamentation as a response (Kadosh and Lopez-Ribot, 2013).

### *In vitro* pharmacological profiling for the detection of potential off-target effects of the leading candidate compound

**Figure 8:**
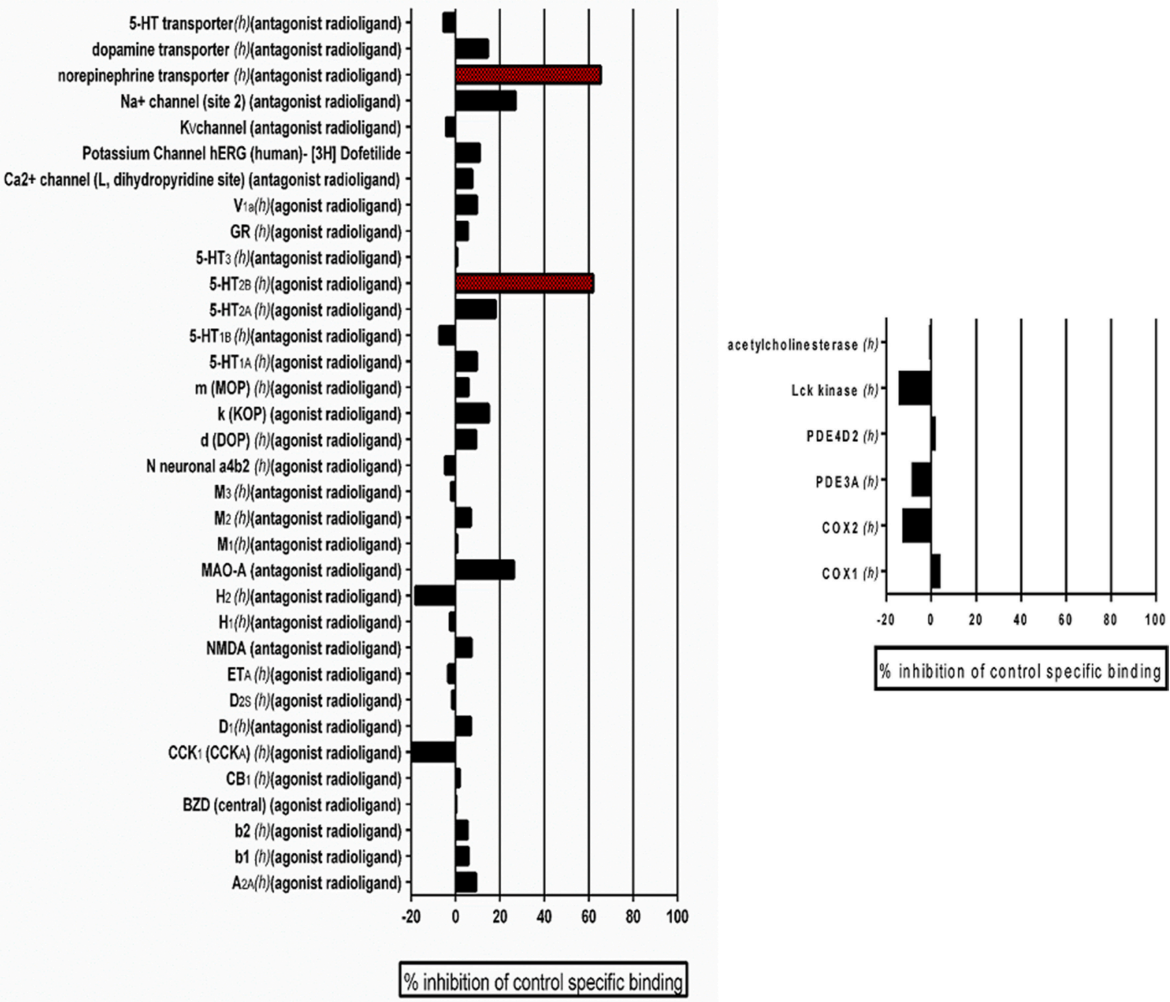
Results of Eurofin Cerep Safety-Screen 44 Panel. *In vitro* pharmacological profiling and assessment of the potential for off-target interactions of the 9029936 leading compound in binding screens (Eurofins Cerep-Panlabs SafetyScreen 44). The compound was screened at a 10 μM concentration in duplicate for its potential to interfere with the binding of native ligands of 44 different receptors, ion channels and enzymes. For receptor assays, compound binding was calculated as percent inhibition of the binding of a radioactively labeled ligand specific for each target. For enzyme assays, the inhibition effect was calculated as percent inhibition of control enzyme activity. Tests were performed in duplicate and results are averages of those two tests.

Previously, we performed standard cytotoxicity studies using HepG2 cell lines (Romo et al., 2017) that pointed to the fact that the lead compound displays a relatively safe profile. In order to expand on potential safety liabilities and identify off-target effects early in the process that could lead to potential undesirable effects and future safety liabilities in the clinic, and to provide additional reassurance for the potential for drug development, as well as guidance for follow-up strategies, we performed an *in vitro* pharmacological profiling of the of the leading 9029936 compound against 44 different targets (mostly human, including G- protein-coupled receptors, ion channels, enzymes and neurotransmitter transporters) (Whitebread et al., 2005; Hamon et al., 2009; Bowes et al., 2012). Results indicated that compound 9029936, when tested at 10 μM concentration (higher than the concentrations needed to inhibit filamentation and biofilm formation) had a relatively clean profile and minimal off-target activities, leading to 50% inhibition or higher in only 2 of the total 44 assays (Figure 8). This results in a promiscuity index (the target hit rate, defined as the percentage of targets giving >50% inhibition at 10 μM) of <5%, which would categorize this compound as “selective” (Whitebread et al., 2005; Hamon et al., 2009). We note that one of the hits is on the 5-hydroxytryptamine (serotonin) receptor 2B (5-HT_2B_), which is linked to cardiac effects, particularly during embryonic development; whereas the other hit is on the norepinephrine transporter, linked to increased heart rate and blood pressure, as well as constipation (Whitebread et al., 2005).

## Discussion

The opportunistic pathogenic fungus *C. albicans* is capable of causing a variety of infections ranging from mucosal to life-threatening systemic candidiasis (Fidel, 1998; Wilson et al., 2002; Hajjeh et al., 2004; Kojic and Darouiche, 2004; Thompson et al., 2010; Kim and Sudbery, 2011; Kullberg and Arendrup, 2015; Allison et al., 2016; McCarty and Pappas, 2016). These infections represent an increasing threat to an ever expanding population of immune- and medically compromised individuals and candidiasis is now the third-to-fourth most frequent nosocomial infection in US hospitals (Wright and Wenzel, 1997; Ramage et al., 2005; Schmiedel and Zimmerli, 2016). Fungal and animal cells are very similar, making antifungal discovery efforts much more arduous than those for antibacterial antibiotics. Unfortunately there is a paucity of antifungal drugs and their efficacy is limited, and as a result clinical outcomes of candidiasis remain far from ideal (Ostrosky-Zeichner et al., 2010; Perfect, 2017). Targeting pathogenetic mechanisms can expand the number of potential targets that can be exploited for antifungal drug discovery, leading to the development of novel anti-virulence approaches to antifungal therapy (Pierce et al., 2015a; Romo et al., 2017; Vila et al., 2017). Filamentation and biofilm formation constitute two of the main virulence factors associated with the ability of *C. albicans* to cause active infection (Mayer et al., 2013). Most recently our group has described a novel series of small molecule compounds with a common biaryl amide core structure that display potent inhibitory activity against *C. albicans* filamentation and biofilm formation (Romo et al., 2017). The leading compound in this series, compound 9029936, represents a candidate for the development of novel anti-virulence therapies against *C. albicans* infections.

In a first series of experiments we demonstrated that besides its activity against the *C. albicans* SC5314 laboratory strain (as expected because of its anti-virulence mode of action), the lead compound proved to be effective against a panel of *C. albicans* clinical isolates, regardless of their underlying molecular mechanism of antifungal drug resistance (i.e., modification in the target enzyme and/or overexpression of efflux pumps) (White, 1997a,b). Likewise, compound 9029936 was also active against *C. albicans* gain of function strains in key transcriptional activators of ergosterol biosynthesis and efflux pumps, all of which are resistant to azole antifungals (Schubert et al., 2008; Flowers et al., 2012). These results underscore the potential of the lead compound as an effective therapeutic against *C. albicans* infections, including those recalcitrant to therapy with current conventional antifungal drugs.

Although *C. albicans* remains the main etiological agent of candidiasis and is responsible for up to 70% of fungal infections, in the last two decades the importance of non-*albicans Candida* species as opportunistic pathogens has emerged (Sullivan et al., 2004, 2005; Silva et al., 2012; Priest and Lorenz, 2015). The high prevalence of non-*albicans Candida* species in disease could also be a reflection of their inherently higher level of resistance to certain antifungal drugs, and/or their increased propensity to develop resistance than *C. albicans* (Silva et al., 2012). In fact, it has been demonstrated that the selective pressure imposed by antifungal treatment can lead to the replacement of *C. albicans* by one of the less common species (Martinez et al., 2002). Overall, infections caused by these non-*albicans Candida* species are often more challenging to detect, diagnose, and treat, and often leads to high morbidity and mortality rates (Criseo et al., 2015). By definition high specificity for the target microorganism is another expected characteristic of an anti-virulence compound, due to virulence factors often being specific to a single microorganism or group of microorganisms (Rasko and Sperandio, 2010). As a result, anti-virulence approaches generally exhibit a narrower window of activity and spectrum of action, as they are only effective against the species displaying the specific virulence factor. For example, our leading compounds exert their activity by inhibiting the morphological transition from yeast to hypha, as well as filamentation-associated biofilm formation. Although these represent the two major pathogenetic mechanisms of *C. albicans*, other *Candida* species display varying degrees in their abilities to filament and form biofilms (Gilfillan et al., 1998; Ramage et al., 2001a; Jabra-Rizk et al., 2004; Brunke and Hube, 2013). As a consequence, we would expect the activity of our leading anti-virulence compound against other non-*albicans Candida* species to differ widely depending on their intrinsic ability to undergo these morphogenetic transitions. Here we expanded our investigations to examine the *in vitro* activity of our leading compounds against different non-*albicans Candida* species including *C. glabrata* (unable to filament), *C. tropicalis* (with limited ability to filament), and *C. dubliniensis* (high filamentation ability, but generally lower than that of *C. albicans* Thompson et al., 2011). Not surprisingly, we observed the highest levels of activity only against *C. dubliniensis*, which is closely related to *C. albicans* with whom it shares a number of phenotypic characteristics, including the ability to filament and form biofilms (Gilfillan et al., 1998; Sullivan and Coleman, 1998; Ramage et al., 2001a). Although the filamentation machinery in *C. dubliniensis* appears to be similar to that of *C. albicans*, but has not been fully characterized (Gilfillan et al., 1998; Martins et al., 2007; Moran et al., 2007; Sullivan and Moran, 2011), the fact that compound 9029936 was able to inhibit *C. dubliniensis* filamentation under all conditions tested reinforces our previous results with *C. albicans* (Romo et al., 2017). Thus, since each medium condition stimulates a specific filamentation pathway, it is likely that the compound does not target any single signaling pathway, but rather interacts with a common component of the filamentation machinery required for filamentation under all conditions, in both *C. albicans* and *C. dubliniensis*. As described above, compound 9029936 exhibits a spectrum of action specific against *C. albicans* and *C. dubliniensis*, but not against *C. glabrata* or *C. tropicalis*, most likely related to the different filamentation capabilities exhibited by the different species. Although this could be perceived as a limitation, it would be expected that this specific activity would exert less stress on the target organism and the host microbiome. Additionally, this narrow activity further validates the anti-virulence nature of the compound.

One distinct possibility given their mode of action, is that anti-virulence agents may be used therapeutically in combination with conventional antibiotics. As described above, combinatorial therapy is widely used for viral and bacterial infections, but it is relatively uncommon in the case of fungal infections (Baddley and Pappas, 2005; Spitzer et al., 2017). Thus, we were interested in testing the activity of our compound together with existing antifungal drugs fluconazole, amphotericin B and caspofungin. We tested the effect of these combinations under all different growth conditions: inhibition of planktonic growth, inhibition of biofilm formation and against preformed biofilms. Although synergy was not widely observed, the leading compound displayed indifference when tested in combination with clinically-used antifungals. This is in contrast to combinations between azoles and echinocandins for which antagonistic effects have previously been described (Bachmann et al., 2003). Together these observations suggest that compound 9029936 could potentially be used successfully together with these antifungals, effectively combining anti-virulence and conventional approaches for the treatment of *C. albicans* infections. Although this compound might be better administered as a prophylactic therapy, based on the drug combination results it could also be used in combinatorial therapy, a strategy which has not been fully adopted in the clinic (Baddley and Pappas, 2005). Moreover, although we have previously demonstrated the efficacy of this compound *in vivo* in the murine model of oral candidiasis which uses immunocompromised mice (Romo et al., 2017), a potential limitation of this type of compounds is that their use could be limited in certain immunosuppressed patients. This is probably where its use in combination could be indicated.

Another main characteristic expected from an anti-virulence compound is an overall lack of effect on growth (Pierce et al., 2015a). Cell viability studies clearly demonstrated that concentrations as high as 40 μM of the lead compound (more than 20 times higher than those required to inhibit filamentation), were unable to kill or arrest cell growth of *C. albicans* cells, confirming that the lead compound is not toxic to fungal cells and acts by specifically disarming the fungus of its virulence factors. Although cytological profiling showed that treatment with the compound results in changes to the cell wall and vacuolar integrity, these effects are likely secondary to the main filamentation deficiency, and do not result in overall growth defects at physiological concentrations tested (40 μM or below). Also, anti-virulence approaches should, at least in theory, prevent the development of resistance since they exert much lower levels of selective pressure as compared to conventional antibiotics, including antifungals (Clatworthy et al., 2007; Rasko and Sperandio, 2010; Ling et al., 2015). Consistent with this prediction, results from serial passage experiments demonstrated that repeated exposure against compound 9029936 did not lead to changes in *C. albicans* susceptibility, which underscores its low potential for fostering resistance.

Besides its inhibitory activities, we have previously reported on the favorable cytotoxicity properties displayed by compound 9029936, underscoring its safety and potential for further development (Romo et al., 2017). *In vitro* pharmacological profiling is increasingly being used earlier in the drug development process to identify potentially undesirable off-target activities and predict clinical adverse events that could jeopardize the development of lead compounds as drug candidates (Bowes et al., 2012). The Eurofins CEREP SafetyScreen 44 panel is recommended by a number of pharmaceutical companies to identify potential pharmacological safety liabilities (Whitebread et al., 2005; Hamon et al., 2009; Bowes et al., 2012). The relatively clean profile and low promiscuity index displayed by compound 9029936 in these tests seems to confirm its potential for further development.

The use of anti-virulence molecules such as 9029936 would expand the alarmingly limited antifungal arsenal. Moreover, targeting pathogenic processes not required for cell survival should also minimize the environmental pressure on the target microorganism, which is the main reason leading to the development of resistance to antifungal agents. Additionally, due to the high specificity of this compound toward *C. albicans* and *C. dubliniensis* filamentation, it would presumably not have negative effects on the host microbiota such as those seen with other broad spectrum antimicrobials.

Altogether, our results confirm that our leading compound acts as a true anti-virulence agent, and confirm that compounds within this series hold potential for the development of novel anti-virulence strategies against candidiasis, including for infections refractory to treatment with conventional antimycotics. These novel approaches are much needed to both enhance and add diversity to the exceedingly limited antifungal armory currently available for the therapy of these devastating infections.

## Author contributions

JR performed most *in vitro* experiments, CP performed initial characterization. ME and C-YH helped with the imaging flow cytometry and conducted the analysis. JR, SS, and JL-R designed follow-up studies. JR, SS, and JL-R wrote and edited the manuscript.

### Conflict of interest statement

The authors declare that the research was conducted in the absence of any commercial or financial relationships that could be construed as a potential conflict of interest.
